# On the Action of Methotrexate and 6-Mercaptopurine on *M. avium* Subspecies *paratuberculosis*

**DOI:** 10.1371/journal.pone.0000161

**Published:** 2007-01-24

**Authors:** Robert J. Greenstein, Liya Su, Vahram Haroutunian, Azra Shahidi, Sheldon T. Brown

**Affiliations:** 1 Laboratory of Molecular Surgical Research, VA Medical Center, Bronx, New York, United States of America; 2 Psychiatry, VA Medical Center, Bronx, New York, United States of America; 3 Microbiology, VA Medical Center, Bronx, New York, United States of America; 4 Infectious Diseases, VA Medical Center, Bronx, New York, United States of America; The Research Institute for Children, United States of America

## Abstract

**Background:**

Clinical improvement in inflammatory bowel disease (IBD) treated with methotrexate and 6-mercaptopurine (6-MP) is associated with a decrease in pro-inflammatory cytokines. This has been presumed to indicate the mechanism of action of methotrexate and 6-MP. Although controversial, there are increasingly compelling data that *Mycobacterium avium* subspecies *paratuberculosis* (MAP) may be an etiological agent in some or all of IBD. We hypothesized that the clinical efficacy of methotrexate and 6-MP in IBD may be to simply inhibit the growth of MAP.

**Methodology:**

The effect on MAP growth kinetics by methotrexate and 6-MP were evaluated in cell culture of two strains each of MAP and *M. avium* using a radiometric (^14^CO_2_ BACTEC®) detection system that quantifies mycobacterial growth as arbitrary “growth index units” (GI). Efficacy data are presented as “percent decrease in cumulative GI” (% −ΔcGI).

**Principal Findings:**

The positive control antibiotic (clarithromycin) has ≥85% −ΔcGI at a concentration of 0.5 µg/ml. The negative control (ampicillin) has minimal inhibition at 64 µg/ml. MAP ATCC 19698 shows ≥80% −ΔcGI for both agents by 4 µg/ml. With the other three isolates, although more effective than ampicillin, 6-MP is consistently less effective than methotrexate.

**Conclusions:**

We show that methotrexate and 6-MP inhibit MAP growth *in vitro*. Each of the four isolates manifests different % −ΔcGI. These data are compatible with the hypothesis that the clinical improvement in patients with IBD treated with methotrexate and 6-MP could be due to treating a MAP infection. The decrease in pro-inflammatory cytokines, thought to be the primary mechanism of action, may simply be a normal, secondary, physiological response. We conclude that henceforth, in clinical studies that evaluate the effect of anti-MAP agents in IBD, the use of methotrexate and 6-MP should be excluded from any control groups.

## Introduction

*Mycobacterium avium* subspecies *paratuberculosis* (MAP) causes Johne's disease [1] in cattle worldwide. Johne's disease is clinically evocative of inflammatory bowel disease (IBD) in humans. The possibility that MAP may be zoonotic [2] is the subject of much interest [3] [4] (& see [5] for review.) Since first seeing our MAP RNA data [6], we have posited in private, in peer reviewing manuscripts [3], [7], [8], and at professional congresses that MAP is the primary and most culpable, potential etiological agent for some or all of IBD.

In the therapy of IBD, (and several other inflammatory diseases) both methotrexate and 6-MP are used because of empirical efficacy, even though their precise mechanism of action is unknown. [9] [10], [11] Their use is attended by clinical improvement that is associated with a decrease in pro-inflammatory cytokines. Consequently, prevailing medical dogma posits that the mode of action of methotrexate and 6-MP, is to decrease the production of pro-inflammatory cytokines, and as a consequence the “inflammatory” response that is the consequence of these cytokines is diminished. This results in a clinical improvement in diseases that are conventionally conceptualized as being primarily “inflammatory.”

Both methotrexate and 6-MP interfere with DNA replication. Methotrexate acts by inhibiting dihydrofolate reductase, folate generation and the consequent production of adenine.[12] The mechanism of action of 6-MP is to substitute for guanine in DNA replication.[12] Because prokaryotes must synthesize their own folic acid, they should be more susceptible to folate inhibition than eukaryotes. It is noteworthy that there are two distinct doses in human clinical use for both methotrexate and 6-MP. Each agent has a “high” dose, (used in to treat reticuloendothelial malignancies [13], [14]) and a “low” dose (used to treat “inflammatory” diseases. [15] [16])

We hypothesized that the clinically relevant mechanism of action of “low” dose methotrexate and 6-MP in the therapy of IBD, may, in whole or part, be due to the inhibition of MAP growth. If this hypothesis is correct, the decrease in the pro-inflammatory cytokines, heretofore considered the primary mechanism of action of these two agents, could simply represent a secondary phenomenon. The observed decrease in pro-inflammatory cytokines could be ascribed to the treatment of the instigating MAP infection. To evaluate this hypothesis we have studied the effect of methotrexate and 6-MP on MAP and other *M. avium* isolates in culture. The effect of methotrexate has been evaluated on *E. coli* [17], 6-MP on *Salmonella typhimurium* [18] and both agents on *M. tb*. [19] To our knowledge, however, this is the first time that these two agents have been evaluated for their effect on MAP.

## Methods

This study was approved by the Research & Development Committee at the VAMC Bronx NY (0720-06-038) and was conducted under the Institutional Radioactive Materials Permit (#31-00636-07).

### Culture

In this study, we used four well-characterized strains of mycobacteria. Two were MAP, a bovine isolate, ATCC 19698 (ATCC Rockville MD) and “Dominic” a human isolate from an individual with Crohn's disease (originally isolated by R. Chiodini [20].) The *M. Avium* subspecies *avium* strains (hereinafter called *M. avium*) were ATCC 25291 (veterinary source) and *M. avium* 101 [21]. Primary cultures were performed in Middlebrook 7H9 medium supplemented 9:1 with ADC (Both Difco. Detroit MI). All media for MAP (liquid and agar plates) were supplemented with 1 µg/ml Mycobactin J (Allied Monitor. Fayette MO.)

The detergent Tween 80 (recommended to prevent mycobacterial clumping) renders clinically resistant strains of MAP inappropriately susceptible to antimicrobials in cell culture. [22] Accordingly in our experiments, Tween 80 was not used. To minimize mycobacterial clumping, one ml of the log phase bacterial culture (∼GI of 500) in 7H12 medium in the BACTEC vial was passaged ≥20 times through a 25 gauge needle [23] on a one ml syringe (Becton-Dickerson Franklin Lakes NJ), added to five ml 7H9 medium supplemented with Mycobactin J, vortexed and left standing at ambient temperature for 30 minutes. Subsequently, only the upper three of the six ml. were used for inoculations. The number of CFU's inoculated was determined by plating serial dilutions of the inoculum onto 7H10 plates (Difco) supplemented for MAP with Mycobactin J (1 µg/ml) and counted when colonies were easily visualized (two to six weeks). At the time of passage, additional aliquots were plated onto blood agar plates to ensure that inocula were not contaminated with non-mycobacterial organisms.

To confirm the identity of the species studied, DNA was obtained from the isolates (High Pure Template Prep. Kit Roche Diagnostics Indianapolis IN), PCR amplified using primers for IS 900 (MAP) [24]and IS 901/2 [25] (*M. avium* & specific subspecies including *silveticum*) as described. [6] Amplicon sizes were determined using 1% agarose gel electrophoresis. DNA sequence analysis was performed commercially, (Genewiz North Brunswick NJ) with sequence comparison performed using BLAST (NLM).

The positive antibiotic control clarithromycin (gift of Abbott Chicago IL) was dissolved in methanol. The negative control antibiotic was ampicillin (Sigma St Louis MO) which was dissolved in water. Methotrexate and 6-mercaptopurine (both Sigma) were dissolved in NaOH at a maximal final concentration of 50 mM (Sigma). Control inocula were performed using the maximum concentration of each diluent. Agents were tested in serial dilutions from a minimum of 0.05 µg/ml to a maximum of 64 µg/ml (see individual Figures). Aliquots of chemicals being evaluated were prediluted, stored at −80°C, thawed, used once and discarded.

Data are presented as cumulative growth index (cGI) units±SD (when necessary, see individual figures). The effect (or lack thereof) of each agent is presented as the percent decrease in cGI units (% −*Δ*cGI), compared to the control cGI of that isolate in the diluent (e.g. methanol or NaOH) that was used for the specific chemical being evaluated. cGI data for each experiment is presented until the day prior to any GI reading exceeding the assay limit of “999” (Table 1). Raw data was archived onto Excel, and the cumulative results transferred to Prism (Graphpad, San Diego CA) for final graphing.

**Table 1:**
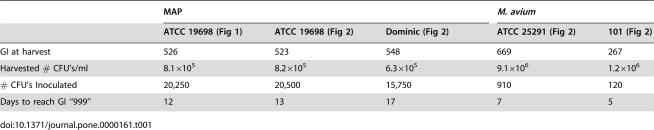
Differences in growth kinetics and consequent length of experiment for each isolate.

	MAP			*M. avium*	
	ATCC 19698 (Fig 1)	ATCC 19698 (Fig 2)	Dominic (Fig 2)	ATCC 25291 (Fig 2)	101 (Fig 2)
GI at harvest	526	523	548	669	267
Harvested # CFU's/ml	8.1×10^5^	8.2×10^5^	6.3×10^5^	9.1×10^6^	1.2×10^6^
# CFU's Inoculated	20,250	20,500	15,750	910	120
Days to reach GI “999”	12	13	17	7	5

## Results

Bacterial quantification must be performed retrospectively. Accordingly, for experimental reproducibility, bacterial passage and harvesting were performed when the GI was ∼500. Quantification show that the CFU's of the *M. avium* isolates are approximately 10 fold higher (∼1×10^7^ CFU's/ml), compared to MAP (∼1×10^6^ CFU's/ml) (Table 1). Because of the difference in growth kinetics, *M. avium* CFU #’s inoculated were ≥10 fold lower than MAP (Table 1).

Both of our MAP isolates (ATCC 19698 & Dominic) were Mycobactin J dependant (data not presented), were IS 900 positive and had ≥99% homology with the GenBank accession NC_002944 of MAP (data not presented). *M. avium* ATCC 25 291 was positive for IS 902 and *M. avium* 101 was negative for both (Data not presented).

**Figure 1:**
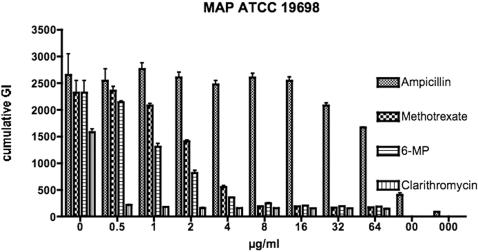
Shown is a bar graph of the cumulative GI data evaluating MAP ATCC 19698. Each drug dilution was studied in duplicate. Error bars are SD. There are three control inoculations, labeled on the abscissa as “0, 00 & 000. The left hand “0” had an equal number of CFU's as in each drug studied. “00”  =  10 & “000”  =  100 fold dilutions. In each control the maximum concentration of diluent used for each agent (methanol for Clarithromycin, water for ampicillin and NaOH for methotrexate and 6-MP) was added. Clarithromycin is most effective and ampicillin is the least effective at decreasing MAP growth. Both methotrexate and 6-MP are as effective as clarithromycin in MAP %−*Δ*cGI at a dose of 4 µg/ml.

In our pivotal study (Figure 1) the positive control antibiotic, clarithromycin exhibits ≥86% −*Δ*cGI at the lowest concentration evaluated (0.5 µg/ml). The negative control antibiotic (the β−lactam, ampicillin) has a minimal effect (21% −*Δ*cGI) at the 32 µg/ml. In contrast, 6-MP has an initial ≥43% −*Δ*cGI starting at 1 µg/ml increasing to ≥84% −*Δ*cGI at 4 µg/ml. Methotrexate has 40% −*Δ*cGI inhibition at 2 µg /ml and ≥75% −*Δ*cGI at 4 µg/ml. (Figure 1.)

**Figure 2:**
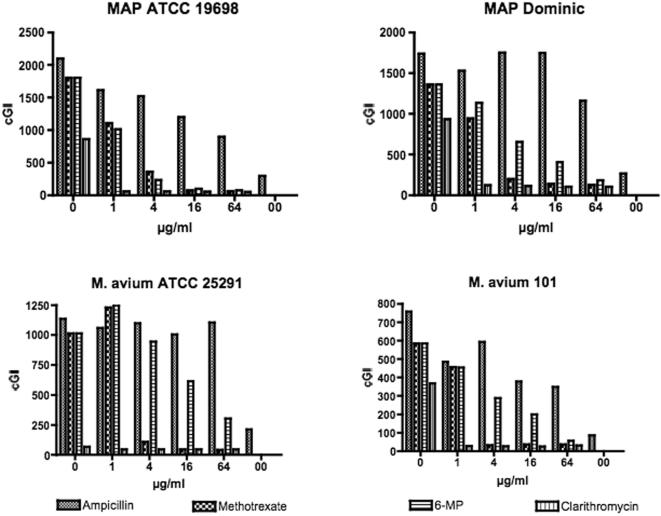
A composite graph of the four bacterial strains studied. “0” is diluent control with an equal CFU inoculum and “00” is a 1∶10 dilution of the water control inoculum. Drug concentrations are indicated on the abscissa. For each isolate, drug dose was studied in singlicate. For all four bacteria, clarithromycin has achieved maximal inhibition by 1 µg/ml. For MAP ATCC 19698, replicating data presented in Figure 1, both methotrexate and 6-MP %−*Δ*GI is the same as for clarithromycin by 4 µg/ml. Note that with the other MAP isolate (Dominic) and both *M. avium* isolates, methotrexate is more effective at a lower dose than is 6-MP. The lower cumulative GI (seen on the ordinate) for the *M. avium* isolates is ascribable to their more rapid growth and earlier reaching the off scale BACTEC GI values of “999.”

We additionally evaluated the effect of methotrexate and 6-MP against two MAP and two *M. avium* isolates (Figure 2). In these studies, the MAP 19698 results replicate the data presented in Figure 1 showing ∼80% −*Δ*cGI inhibition at 4 µg/ml for both 6-MP and clarithromycin. In contrast, MAP Dominic shows less susceptibility to 6-MP (41% −*Δ*cGI at 4 µg/ml) compared to MAP 19698 (84%−*Δ*GI at 4 µg/ml). Both *M. avium* isolates show less susceptibility to 6-MP than to methotrexate (Figure 2). The diluent control inoculum for the *M. avium* ATCC 25291 appears to exhibit completely inhibited growth (Figure 2: Bottom left graph, left hand column.) However, over the following two days this methanol control entered log phase growth, whereas the vials at every clarithromycin dose continued to show no evidence of growth (data not presented.)

## Discussion

The efficacy of both methotrexate and 6-MP in the therapy of IBD is uncontested. Prevailing dogma accepts that the decrease in pro-inflammatory cytokines that attends their use is responsible for their beneficial effect. In this study we show that both methotrexate and 6-MP inhibit the growth kinetics of MAP. In the event that IBD is eventually accepted as being due to a MAP infection, our data are compatible with our hypothesis that methotrexate and 6-MP may be impairing MAP growth. If so, the decrease in pro-inflammatory cytokines could simply be an appropriate physiological response to their antibiotic-like activity.

We additionally show that there is a variation in response of the four different isolates to our tested agents. Three of the four isolates are more sensitive to methotrexate than to 6-MP. These observations need to be further evaluated in multiple isolates from a variety of individuals and clinical settings where development of MAP resistance may be responsible for a clinical deterioration. We conclude that antibiotic susceptibility testing will probably be indicated for putative MAP infections, just as they are for other (myco) bacterial infections.

As is conventional with antibiotic susceptibility studies, we compared agents on an equal weight basis. However, methotrexate (MW 450) is a much larger molecule than 6-MP (MW 170) with a molar ratio of ∼3∶1. Thus, in comparison to 6-MP on a molar basis, methotrexate is an even more potent inhibitor of growth than our data indicate. Additionally, a simple extrapolation of our data to a comparison with conventional “antibiotics” therapy is difficult. The dosages of methotrexate and 6-MP in clinical use have not been titrated according to standard antibiotic conventions. Dosage has been individualized, influenced by such factors as hematological toxicity and patient tolerance.

There is a remarkable discrepancy between the doses of methotrexate and 6-MP, used to treat different diseases, that merit discussion. Each agent has a “high” dose, (used in to treat reticuloendothelial malignancies [13], [14]) and a “low” dose (used to treat “inflammatory” diseases. [15] [16]) For methotrexate the antineoplastic dose may be 1500–5000 mg M^2^ by IV infusion over 2–24 hours (for a 70 kg man this could equate to 7500 mg in 24 hours.)[26] In contrast, for IBD a typical dose is 25 mg PO or IM weekly. We suggest that this 300-fold disparity may reflect the difference between treating a eukaryotic reticuloendothelial malignancy and a prokaryotic mycobacterial (specifically we hypothesize in the case of IBD a MAP) infection.

Our data are compatible with the thesis, but do not conclusively prove, that MAP may be zoonotic. Corroboration of our data will be necessary and multiple additional studies, both basic and clinical need to be performed. However, we suggest that, as a consequence of our observations antecedent clinical studies that have evaluated anti-MAP agents need to be reevaluated and that henceforth such studies will need to exclude methotrexate and 6-MP from “control” or placebo subjects.

### Conclusions and recommendations

We show that both methotrexate, as well as 6-MP, interfere with the growth of MAP, an organism that may be the etiological factor for some, or all of IBD. Some of the implications of these observations are discussed.
